# P121: Clean Hands project: seven years of supporting hand hygiene compliance

**DOI:** 10.1186/2047-2994-2-S1-P121

**Published:** 2013-06-20

**Authors:** AFV Tipple, JLU Spagnoli, ZCP Neves, JEM Santos, FCR Cesar, JPDA Trindade, KCDO Batista, KM Mendonça

**Affiliations:** 1 Faculdade de Enfermagem, Universidade Federal de Goiás, Goiânia, Brazil

## Introduction

Evidence of the role of hand hygiene (HH) in reducing morbidity and mortality rates associated with infection stimulated the creation of the "Clean Hands" project.

## Objectives

To present the experiences and outcomes of the project "Clean Hands" that, over the past seven years, has developed a program to encourage hand hygiene.

## Methods

The project is based at the Center for Studies and Nursing Research for the Prevention and Control of Healthcare Associated Infections (NEPIH) at the School of Nursing, Federal University of Goiás/Brazil, and has developed activities to encourage HH since 2006 within the healthcare establishment, with professionals, academics, patients and caregivers; municipal daycare centers, with children, parents, and workers; and in scientific events, with academics and healthcare professionals. Strategies that have incentivized compliance with HH procedures: informative stylized banners depicting HH; educational brochures, a song parody CD, demonstration of proper HH technique, using poster paints on children’s hands; a puppet theater and face-to-face discussions about the importance of, obstacles to, and benefits of HH. Annually, the project hosts a festival of parodies about HH, called "CANTAFEN", which brings together academics and healthcare professionals.

## Results

The project’s day-to-day operations are normally run by five students, supported by the other members of NEPIH, currently 33 staff members (faculty, undergraduate and graduate), participating in the activities. The project has performed about 180 campaigns (45 were for children) reaching approximately 8.000 people.

## Conclusion

Participation in the project has contributed to development skills and competencies with regard to the implementation of health promotion strategies with different audiences and requires that students constantly stay up to date on the subject. The festival of parodies has helped to empower its members to conduct scientific and cultural events and promote emphasis of the subject in a playful manner. Although the nature of the project as an extension program hinders the assessment of the impact on adherence to HH, it is possible to infer a much larger number of beneficiaries than those directly benefitted by the actions of the group.

## Disclosure of interest

None declared

